# J-shaped association of the triglyceride glucose-body mass index with new-onset diabetes

**DOI:** 10.1038/s41598-024-64784-0

**Published:** 2024-06-16

**Authors:** Qincheng Qiao, Kai Liang, Chuan Wang, Lingshu Wang, Fei Yan, Li Chen, Xinguo Hou

**Affiliations:** 1 Department of Endocrinology and Metabolism, Qilu Hospital of Shandong University, Jinan, Shandong 250012 People’s Republic of China; 2 Institute of Endocrine and Metabolic Diseases of Shandong University, Jinan, 250012 People’s Republic of China; 3 Key Laboratory of Endocrine and Metabolic Diseases, Shandong Province Medicine & Health, Jinan, People’s Republic of China; 4 Jinan Clinical Research Center for Endocrine and Metabolic Diseases, Jinan, People’s Republic of China; 5 The First Clinical Medical College, Cheeloo College of Medicine, Shandong University, Jinan, People’s Republic of China

**Keywords:** Endocrinology, Risk factors

## Abstract

The triglyceride glucose-body mass index (TyG-BMI) is a convenient and clinically significant indicator of insulin resistance. This study aims to investigate the correlation between TyG-BMI and the onset of new-onset diabetes and determine an optimal reflection point for TyG-BMI. An analysis was conducted on 1917 participants from the risk evaluation of cancers in Chinese diabetic individuals: a lONgitudinal (REACTION) study. Participants were categorized based on their TyG-BMI, and the relationship between TyG-BMI and the incidence of new-onset diabetes was explored through logistic regression models, smoothed curve fitting with restricted cubic spline, and a two-piecewise logistic regression model. The mean age of the participants was 57.60 ± 8.89 years, with 66.5% being females. The mean TyG-BMI was 223.3 ± 32.8. Ultimately, 137 individuals (7.1%) progressed to diabetes after three years. After adjusting for covariates, TyG-BMI exhibited a positive correlation with new-onset diabetes (odd ratios (OR) for each standard deviation increase = 1.330, 95% CI 1.110–1.595). The relationship between TyG-BMI and new-onset diabetes was non-linear, with a inflcetion point at 202.9. This study reveals a positive non-linear relationship between TyG-BMI and the risk of new-onset diabetes in Chinese middle-aged and elderly individuals. When TyG-BMI exceeds 202.9, there is a significantly heightened risk of new-onset diabetes. These findings offer valuable insights for preventing new-onset diabetes.

## Introduction

Over the past 3 decades, the global incidence of diabetes has doubled, solidifying its position as the ninth leading cause of death^1^. The escalating prevalence of diabetes poses a substantial public health concern worldwide, especially in developing countries^2^. Epidemiological studies reveal that approximately 11% of the Chinese population is affected by diabetes, with a significant portion remaining undiagnosed^3^. Consequently, an effective indicator is paramount to predicting and identifying individuals at a high risk of diabetes.

The prediction of diabetes occurrence relies on various relevant indicators, with insulin resistance (IR) being a pivotal factor in the transition from normal individuals to diabetes^4,5^. The hyperinsulinemic-euglycemic clamp test currently stands as the diagnostic gold standard for IR. However, its invasive nature, time-consuming process, high cost, and complexity make widespread implementation in clinical settings or efficient population screening challenging^6^. Therefore, identifying new, more convenient, and easily measurable predictive indicators is imperative for recognizing individuals at a high risk of diabetes.

Recently, several new indicators for predicting the onset of diabetes have emerged, such as the visceral adiposity index (VAI)^7^ and triglyceride glucose (TyG) index^8^. The triglyceride glucose-body mass index (TyG-BMI) is a recently developed reliable surrogate for IR, comprising three easily measurable parameters: BMI, triglycerides (TG), and fasting plasma glucose (FPG). Recent epidemiological studies have highlighted the excellent value of TyG-BMI in diagnosing diabetes and other chronic diseases by effectively identifying IR^6,9–11^. While previous research has assessed the linear relationship between TyG-BMI and the incidence of new-onset diabetes, the optimal range of TyG-BMI in the general population remains undetermined.

To address the issues, we conducted a retrospective cohort study to comprehensively assess the relationship between TyG-BMI and diabetes. Specifically, we aimed to investigate the potential non-linear relationship between TyG-BMI and progression to diabetes and determine the optimal control range of TyG-BMI. This study provides new insights for more precise diabetes prevention strategies.

## Methods

### Study population and design

The current investigation constitutes a segment of the baseline and 3-year follow-up examinations conducted as part of the risk evaluation of cancers in Chinese diabetic individuals: a longitudinal (REACTION) study^12,13^. In the baseline survey of 2012, we scrutinized 10,028 participants (6458 women) aged between 40 and 90 years from four urban communities (one in Jinan City and three in Jining City) in Shandong Province, China. Subsequently, in 2015, we conducted a 3-year follow-up, representing the first visit post-baseline survey.

**Figure 1:**
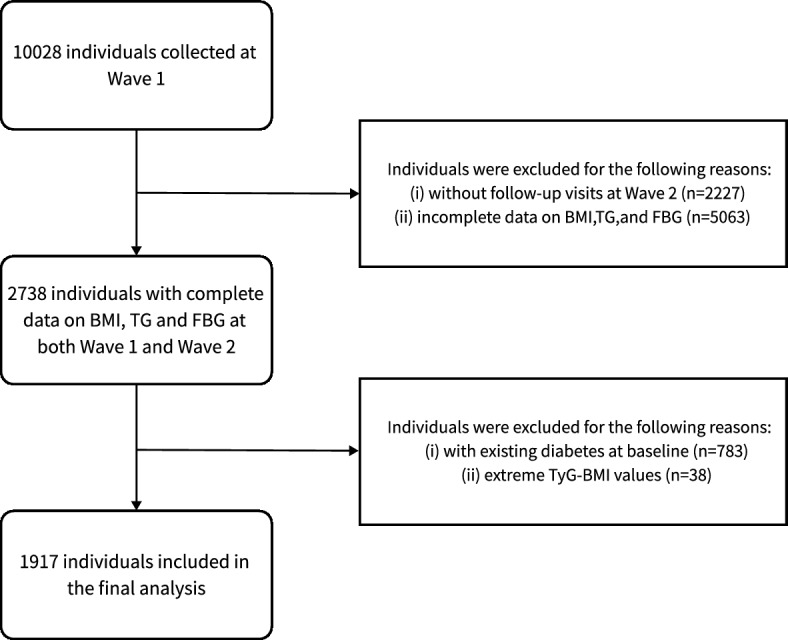
Flowchart depicting the progression of study participants.

The follow-up included 4778 subjects who participated in on-site follow-ups, undergoing repeat measurements of HbA1c and oral glucose tolerance tests (OGTT). Additionally, it encompassed 2864 subjects who underwent telephone surveys, 159 subjects who did not survive until the follow-up, and 2227 subjects who lost to follow-up, resulting in a follow-up rate of 77.8%. Of the 7801 subjects who participated in the follow-up, we first excluded 5063 subjects with missing BMI, TG, or FPG. After that, we further excluded 783 subjects with diabetes at baseline and 38 subjects with extreme TyG-BMI values. Finally 1917 subjects were included in the study (Fig. 1).

### Data collection and clinical evaluation

All researchers involved in both surveys underwent extensive training related to the research questionnaire and outcome measurements before the commencement of the investigations. Demographic characteristics and lifestyle data were collected through face-to-face interviews utilizing standardized questionnaires. Human body measurements were obtained, including height, weight, and blood pressure (BP). The body mass index (BMI) was calculated as weight (kg) divided by the square of height (m^2^).

Three consecutive blood pressure measurements were obtained at 1-min intervals using the right arm, and the average of these three measurements was utilized for analysis. Fasting blood samples were collected in the morning after at least a 10-h fast and 2 h after ingesting a 75 g oral glucose load for the oral glucose tolerance test (OGTT). Glucose levels were determined using an automatic clinical chemistry analyzer employing the glucose oxidase method. Glycated hemoglobin (HbA1c) was measured using an automated glycohemoglobin analyzer (VARIANT, Bio-Rad, USA™) through ion-exchange high-performance liquid chromatography. All clinical measurements were performed following the manufacturer's instructions.

### Definitions and diagnostic criteria

In our study, the exposure variable is TyG-BMI, calculated using the formula: TyG-BMI = BMI * Ln [TG (mg/dL) × FPG (mg/dL)/2]. Diabetes, in this study, is defined according to the American Diabetes Association (ADA) guidelines. Diabetes is defined as FPG ≥ 7.0 mmol/L and/or OGTT ≥ 11.1 mmol/L and/or glycosylated hemoglobin (HbA1c) ≥ 6.5%^14^. Hypertension is defined as an average systolic blood pressure (SBP) ≥ 140 mm Hg and/or an average diastolic blood pressure (DBP) ≥ 90 mm Hg or the use of antihypertensive medication. Chronic kidney disease (CKD) is defined as a glomerular filtration rate (GFR) < 90 mL/min * 1.73 m^2^ according to the Kidney Disease Outcomes Quality Initiative (K/DOQI) guidelines^15^. Alcohol consumption was defined in terms of the question “Do you drink alcohol now?” and the question “Have you drank alcohol in the past?” in the question “Do you drink alcohol now?” and the question “Have you drank alcohol in the past? Smoking was defined as an affirmative response to the questions “Do you smoke now?” and the question “Have you smoked in the past?” in the question “Do you smoke now?” and the question “Have you smoked in the past?”. Postprandial glucose was defined as a meal of 75 g of glucose on an empty stomach followed by a venous blood draw 2 h after the meal to measure blood glucose. Family history of diabetes was defined as diabetes mellitus in any of the subject's father, mother, grandmother, grandfather, maternal grandmother, or maternal grandfather.

### Statistical analysis

We conducted statistical analyses using Python 3.8.8 and R 4.2.1, with a two-sided *P* < 0.05 significance level. Baseline variables were described by grouping and summarizing them based on the quartiles of TyG-BMI. Continuous variables with a normal distribution are presented as mean ± standard deviation, while categorical variables are expressed as numbers (proportions). One-way analysis of variance (ANOVA) for continuous variables and Pearson's chi-square test for categorical variables were employed to assess the differences in baseline characteristics among the four TyG-BMI groups defined by quartiles.

Investigating the relationship between TyG-BMI and the diabetes outcome involved three primary steps. In step 1, we analyzed logistic regression, constructing a regression model with no adjusted covariates (Model 1) and three multivariable regression models with stepwise adjustments. In Model 1, we adjusted for demographic factors, including gender and age. Model 2 further considered potential lifestyle impacts, such as smoking and alcohol consumption. Model 3, built upon Model 2, included hypertension, chronic kidney disease and family history of diabetes adjustments. The correlation between TyG-BMI and the diabetes outcome was assessed using quartiles, with the quartile having the lowest incidence rate serving as the reference group.

We also calculated the E-value to quantify the degree of association between unadjusted confounders and regression outcomes. In step 2, to illustrate the potential non-linear relationship between TyG-BMI and the diabetes outcome, we employed a four-node restricted cubic spline (RCS) within logistic regression to fit smoothed curves. If nonlinearity is detected, then it is solved by generating a series of sequences of TyG-BMI values, substituting them into the fitted smoothed curves, and obtaining the TyG-BMI value corresponding to the extremes of all solutions, which is the corresponding reflection point. And after this, the threshold effects on both sides of the reflection point were analyzed using the likelihood ratio test. In step 3, subgroup analyses were conducted by grouping the population based on gender, age, and BMI to explore whether the association between TyG-BMI and the diabetes outcome was specific to these common population phenotypes.

### Ethics approval and informed consent statement

The study protocol received approval from the institutional review board at the Department of Endocrinology and Metabolic Disease, Ruijin Hospital, Shanghai Jiaotong University School of Medicine. The investigations were carried out in accordance with the Declaration of Helsinki. Written, informed consent was obtained from each study participant.

## Results

### Baseline characteristics of study participants

Following population selection, our study comprised 1917 participants, including 1275 females (66.5%). The average age was 57.6 ± 8.9 years, and the mean TyG-BMI was 223.3 ± 32.8. At three years follow-up, 137 individuals (7.1%) progressed to diabetes.

Participants were grouped based on the quartiles of TyG-BMI, with TyG-BMI values for each quartile range as follows: < 199.7, 199.9–221.6, 221.6–246.0, ≥ 246.0. Participants with higher TyG-BMI were more likely to be male or older. They often exhibited higher levels of FPG, postprandial glucose, HbA1c, TG, BMI, SBP, and DBP and lower estimated glomerular filtration rate (eGFR). All these differences were statistically significant (*P* < 0.05) (Table 1). The results of the comparison of the differences between each of the two quartile groups are in sTable 1 of the Supplementary Material.

**Table 1:** Characteristics of the study population based on quartiles of TyG-BMI.

	All	Q1 (≤ 199.75)	Q2 (199.75 to 221.57)	Q3 (221.57 to 245.97)	Q4 (> 245.97)	*P* value
N (%)	1917	480 (25.04)	479 (24.99)	479 (24.99)	479 (24.99)	
Age, years, mean ± SD	57.60 ± 8.88	55.57 ± 9.46	57.24 ± 8.76	58.82 ± 8.44	58.77 ± 8.47	< 0.001
Gender, n (%)						0.001
Male	642 (33.49)	134 (27.92)	148 (30.90)	183 (38.20)	177 (36.95)	
Female	1275 (66.51)	346 (72.08)	331 (69.10)	296 (61.80)	302 (63.05)	
BMI, kg/m^2^, mean ± SD	25.88 ± 3.17	22.27 ± 1.63	24.80 ± 1.26	26.76 ± 1.44	29.69 ± 2.11	< 0.001
Smoking, n (%)						0.006
No	1500 (78.25)	397 (82.71)	384 (80.17)	357 (74.53)	362 (75.57)	
Yes	417 (21.75)	83 (17.29)	95 (19.83)	122 (25.47)	117 (24.43)	
Alcohol consumption, n (%)						0.014
No	1369 (71.41)	369 (76.88)	342 (71.40)	327 (68.27)	331 (69.10)	
Yes	548 (28.59)	111 (23.12)	137 (28.60)	152 (31.73)	148 (30.90)	
Systolic blood pressure, mmHg, mean ± SD	139.18 ± 25.04	132.40 ± 18.36	137.01 ± 19.85	142.79 ± 36.34	144.52 ± 19.19	< 0.001
Diastolic blood pressure, mmHg, mean ± SD	79.99 ± 11.49	76.65 ± 10.98	79.14 ± 11.59	81.29 ± 11.49	82.87 ± 10.96	< 0.001
Fasting plasma glucose, mmol/L, mean ± SD	5.32 ± 0.57	5.18 ± 0.55	5.30 ± 0.56	5.36 ± 0.59	5.45 ± 0.57	< 0.001
Postprandial blood glucose, mmol/L, mean ± SD	5.70 ± 1.52	5.41 ± 1.34	5.62 ± 1.43	5.68 ± 1.58	6.07 ± 1.64	< 0.001
Glycosylated hemoglobin, %, mean ± SD	5.76 ± 0.37	5.63 ± 0.34	5.76 ± 0.35	5.82 ± 0.36	5.83 ± 0.39	< 0.001
eGFR, mL/min per 1.73 m^2^, mean ± SD	95.97 ± 10.03	98.25 ± 10.15	96.77 ± 9.31	94.30 ± 10.12	94.54 ± 9.99	< 0.001
Chronic kidney disease, n (%)						< 0.001
No	1476 (77.00)	397 (82.71)	382 (79.75)	358 (74.74)	339 (70.77)	
Yes	441 (23.00)	83 (17.29)	97 (20.25)	121 (25.26)	140 (29.23)	
Hypertension, n (%)						< 0.001
No	983 (51.28)	322 (67.08)	257 (53.65)	222 (46.35)	182 (38.00)	
Yes	934 (48.72)	158 (32.92)	222 (46.35)	257 (53.65)	297 (62.00)	
Family history of diabetes, n (%)						0.014
No	1790 (93.38)	444 (92.50)	453 (94.57)	458 (95.62)	435 (90.81)	
Yes	127 (6.62)	36 (7.50)	26 (5.43)	21 (4.38)	44 (9.19)	
Outcome diabetes, n (%)^a^						0.001
No	1780 (92.85)	454 (94.58)	458 (95.62)	438 (91.44)	430 (89.77)	
Yes	137 (7.15)	26 (5.42)	21 (4.38)	41 (8.56)	49 (10.23)	

^a^Outcome diabetes was defined as a subject who did not have diabetes at baseline but developed diabetes at follow-up.

### Association between TyG-BMI and diabetes

In populations stratified into four groups based on the TyG-BMI quartiles, participants in the second quartile (Q2) exhibited the lowest final diabetes incidence. In contrast, participants in the fourth quartile (Q4) demonstrated a significantly increased incidence compared to those in the second quartile (Q2). In the regression model treating TyG-BMI as a continuous variable, for each standard deviation increase in TyG-BMI, the risk of diabetes in the population increased by 1.33 times (95% confidence interval 1.110–1.595, with an E value of 1.103). Subsequently, in the regression model treating TyG-BMI quartiles as categorical variables with Q2 as the reference group, the diabetes risk in the populations with outcomes increased by 1.359 times (95% confidence interval 0.749–2.491), 1.895 times (95% confidence interval 1.109–3.329), and 2.387 times (95% confidence interval 1.418–4.149) for Q1, Q3, and Q4, respectively. These regression results suggest a potential non-linear relationship between TyG-BMI and diabetes, indicating that both low and high TyG-BMI levels may elevate the risk of diabetes incidence (Table 2).

**Table 2:** Association of TyG-BMI with diabetes.

	OR (95%CI), *P* value			
	Non-adjusted Model	Model I	Model II	Model III
TyG-BMI (per SD increase)	1.363 (1.148–1.618) < 0.001	1.311 (1.099–1.565), 0.003	1.309 (1.097–1.563), 0.003	1.330 (1.110–1.595), 0.002
TyG-BMI (quartiles, Q1 as reference)				
Q1	Ref	Ref	Ref	Ref
Q2	0.801 (0.440–1.441), 0.460	0.746 (0.408–1.348), 0.333	0.737 (0.403–1.332), 0.314	0.736 (0.401–1.335), 0.315
Q3	1.635 (0.989–2.748), 0.058	1.404 (0.844–2.374), 0.196	1.389 (0.834–2.350), 0.212	1.395 (0.835–2.370), 0.208
Q4	1.990 (1.225–3.302), 0.006	1.738 (1.064–2.898), 0.030	1.720 (1.053–2.870), 0.033	1.756 (1.065–2.955), 0.030
TyG-BMI (quartiles, Q2 as reference)				
Q1	1.249 (0.694–2.273), 0.460	1.341 (0.742–2.451), 0.333	1.358 (0.751–2.484), 0.314	1.358 (0.749–2.490), 0.315
Q2	Ref	Ref	Ref	Ref
Q3	2.042 (1.201–3.571), 0.010	1.883 (1.102–3.306), 0.023	1.886 (1.104–3.312), 0.023	1.895 (1.109–3.330), 0.022
Q4	2.485 (1.486–4.295), 0.001	2.330 (1.388–4.041), 0.002	2.336 (1.391–4.051), 0.002	2.385 (1.416–4.149), 0.001
P-trend	< 0.001	0.004	0.004	0.003

Model 1: adjusted for gender and age.

Model 2: adjusted for gender, age, alcohol consumption and smoking.

Model 3: adjusted for gender, age, alcohol consumption, smoking, chronic kidney disease, hypertension and family history of diabetes.

**Figure 2:**
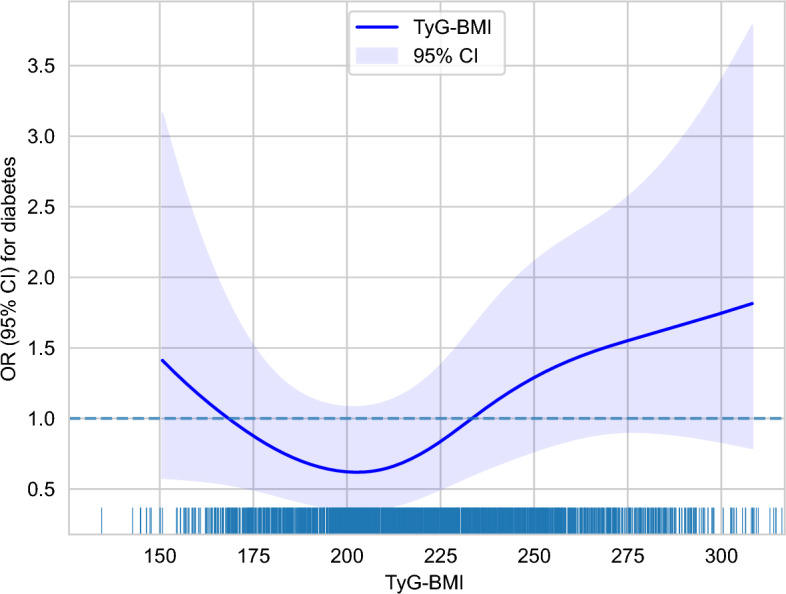
The non-linear relationship between TyG-BMI and the risk of diabetes in participants. We employed a logistic regression model with cubic spline functions to assess the relationship between TyG-BMI and diabetes risk. The results indicated a non-linear association, with the inflection point of the TyG-BMI ratio identified as 202.9.

Smoothed curves were fitted using RCS within logistic regression to investigate the non-linear relationship between TyG-BMI and diabetes further (Fig. 2). The results revealed a J-shaped association between TyG-BMI and diabetes, with a non-linear *P* value of 0.0397. The inflection point for TyG-BMI was determined to be 202.9, and subsequent threshold effect analysis indicated that when TyG-BMI was below 202.9, an increase in TyG-BMI was negatively correlated with diabetes incidence (for every unit increase in TyG-BMI, OR = 0.986, 95% CI 0.969–1.006). However, when TyG-BMI was above 202.9, an increase in TyG-BMI was positively correlated with diabetes incidence (for every unit increase in TyG-BMI, OR = 1.014, 95% CI 1.007–1.021) (Table 3). Likelihood ratio tests comparing segmented and continuous regression models showed a log-likelihood value of − 468.22 for the segmented model and − 470.74 for the continuous model, with a chi-square test *P* value of 0.0247, indicating a superior fit for the segmented model.

**Table 3:** Threshold effect analyses of TyG-BMI on the risk of diabetes using two-piecewise regression models.

TyG-BMI	Non-adjusted Model		Adjusted model	
	OR (95% CI)	*P* value	OR (95% CI)	*P* value
< 202.9	0.991 (0.973–1.011)	0.349	0.986 (0.969–1.006)	0.157
≥ 202.9	1.014 (1.006–1.020)	< 0.001	1.014 (1.007–1.021)	< 0.001

Adjusted model: adjusted for gender, age, alcohol consumption, smoking, chronic kidney disease, hypertension and family history of diabetes.

### Stratified analyses by potential effect modifiers

Subgroup analyses were conducted for different populations based on gender, age, and BMI to explore whether the relationship between TyG-BMI and diabetes exhibited specificity within these common population phenotypes (Table 4). In the results of subgroup analysis, we observed that in males (OR = 1.011, 95% CI 1.002–1.020) compared to females (OR = 1.008, 95% CI 1.000–1.015), there was a stronger positive correlation between TyG-BMI and diabetes. Additionally, in the age group of 40–60 years, the positive correlation between TyG-BMI and diabetes was stronger than in the population aged 60 and above.

**Table 4:** Subgroup analysis evaluating the effect of TyG-BMI on the outcome of diabetes.

Subgroup	No. of participants (percent, %)	OR (95% CI), *P* value	*P* for interaction
Age (years)			0.673
40–60	1171 (61.09%)	1.009 (1.001–1.017), 0.021	
≥ 60	746 (38.91%)	1.008 (1.000–1.016), 0.060	
Sex			0.554
Male	642 (33.49%)	1.011 (1.002–1.020), 0.019	
Female	1275 (66.51%)	1.008 (1.000–1.015), 0.040	
BMI (kg/m^2^)			0.456
< 24	554 (28.90%)	0.987 (0.964–1.011), 0.290	
24–27.9	894 (46.64%)	1.013 (0.997–1.029), 0.108	
≥ 28	469 (24.47%)	1.009 (0.994–1.025), 0.235	

Models adjusted for the same covariates as in model 3 (Table 2), except for the stratification variable.

Results from interaction tests indicated that all *P* values were greater than 0.05, suggesting no significant variables affecting the relationship between TyG-BMI and diabetes regarding gender, age, and BMI.

## Discussion

In this retrospective cohort study involving Chinese individuals aged 40 and above, our findings reveal a noteworthy association between TyG-BMI levels and the risk of incident diabetes. The observed J-shaped pattern in this association indicates that high TyG-BMI level is linked to an increased risk of new-onset diabetes, suggesting potential harm at extremes of TyG-BMI. Furthermore, we pinpointed the inflection point of this association at TyG-BMI at 202.9. Above this threshold, higher TyG-BMI values were associated with an elevated risk of new-onset diabetes. Therefore, maintaining TyG-BMI around 202.9 may be beneficial in reducing the risk of diabetes incidence in the population.

Although the molecular and biological mechanisms connecting TyG-BMI to diabetes remain unclear, there is a potential association with IR. IR is a pivotal factor in the onset and progression of diabetes^16^. Increased glucose concentrations may lead to elevated levels of reactive oxygen species, exerting toxic effects on pancreatic β-cells^17^. Elevated intracellular TG levels may contribute to IR^18^, and an excess of TG in pancreatic cells may disrupt β-cell function^17^. BMI, commonly used to assess the risk of obesity and metabolic diseases, is also associated with an increased risk of developing diabetes^19^. The potential mechanism underlying the relationship between TyG-BMI and diabetes risk may involve the interplay of FPG, TG, and BMI concerning IR.

The onset of diabetes can be predicted by a number of indicators, including basic anthropometric indicators^20^ such as BMI, waist circumference (WC), waist-to-height ratio (WHtR), waist-to-hip ratio (WHR), and hip circumference (HC), as well as the visceral obesity index (VAI), based on both anthropometric and laboratory parameters, and lipid accumulation products (LAP), based on the combination of TG and WC^10^. Whereas insulin resistance (IR) is present before the diagnosis of diabetes and is a key mediator in its pathogenesis. Although hyperinsulin- normoglycemic clamp is the gold standard for measuring IR, it is not suitable for clinical practice due to its invasive and complex examination procedure^6^. And although the HOMA-IR is currently the most widely used non-invasive measure in clinical practice, the method is not ideal for use in patients with impaired β-cell function and insulin therapy^10^. The TyG index has been shown to outperform the HOMA-IR in Chinese patients with type 2 diabetes mellitus^21^.The development of a new obesity-related parameter, TyG-BMI, in recent years has gone even further by incorporating BMI, a classic and simple index widely used to assess obesity. and simple indicator. It has been shown that the predictive value of TyG-BMI for the risk of diabetes mellitus is better than that of TyG index alone^10^. However, previous research has primarily examined the linear relationship between TyG-BMI and incident diabetes, neglecting or not detecting any non-linear associations^6,9,11^. Therefore, investigating the non-linear relationship between TyG-BMI and incident diabetes is essential to determine an optimal control range.

The strength of our study is that it provides new perspectives and tools for public health and clinical practice by revealing the nonlinear relationship between the TyG-BMI index and diabetes risk. Also, clinicians can use this index to personalize treatment and screening,. For those patients whose TyG-BMI index is above the inflection point, lifestyle interventions or medications can be taken more aggressively.

Our study also has some limitations. First, our study population included only middle-aged and older Chinese individuals aged 40 years and older, so caution is needed in generalizing our findings to other populations. Second, the design of the retrospective cohort study limited our ability to determine causality. Therefore, we were unable to determine a causal relationship between TyG-BMI and the incidence of new-onset diabetes. Finally, the relatively short follow-up period of our study, the small number of new-onset diabetes cases in the final population, and the possibility that lost visits and incomplete data may have led to underrepresentation of the sample and introduction of bias in the final analysis constitute additional limitations.

## Conclusion

Our study reveals that excessively high TyG-BMI level is associated with an increased risk of incident diabetes. A J-shaped curve of TyG-BMI is evident in the overall population and subgroups based on gender, age, and BMI, with the inflection point at 202.9. These findings emphasize the importance of maintaining TyG-BMI within a targeted range to effectively reduce the risk of diabetes incidence across diverse demographic groups.

### Supplementary Information

Supplementary Information.

## Data Availability

The data used to support the findings of this study are available from the corresponding author upon request.
