# Supplementary material for: J-shaped association of the triglyceride glucose-body mass index with new-onset diabetes
Source: Sci Rep. 2024 Jun 16;14:13882. doi: 10.1038/s41598-024-64784-0 (PMC11180648; doi:10.1038/s41598-024-64784-0)
Supplement: Supplementary file 1 — Supplementary Information. [file 41598_2024_64784_MOESM1_ESM.docx]

sTable 1. Significance of differences in indicators between the two quartile groups.

| Feature | Q1 vs Q2 | Q1 vs Q3 | Q1 vs Q4 | Q2 vs Q3 | Q2 vs Q4 | Q3 vs Q4 |
| --- | --- | --- | --- | --- | --- | --- |
| Age | 0.005 | ＜0.001 | ＜0.001 | 0.005 | 0.006 | 0.93 |
| Gender | 0.312 | ＜0.001 | 0.003 | 0.017 | 0.048 | 0.689 |
| BMI | ＜0.001 | ＜0.001 | ＜0.001 | ＜0.001 | ＜0.001 | ＜0.001 |
| Smoking | 0.312 | 0.002 | 0.007 | 0.037 | 0.087 | 0.709 |
| Alcohol consumption | 0.053 | 0.003 | 0.007 | 0.292 | 0.437 | 0.781 |
| Systolic blood pressure | ＜0.001 | ＜0.001 | ＜0.001 | 0.002 | ＜0.001 | 0.356 |
| Diastolic blood pressure | ＜0.001 | ＜0.001 | ＜0.001 | 0.004 | ＜0.001 | 0.029 |
| Fasting plasma glucose | ＜0.001 | ＜0.001 | ＜0.001 | 0.082 | ＜0.001 | 0.017 |
| Postprandial blood glucose | 0.023 | 0.005 | ＜0.001 | 0.523 | ＜0.001 | ＜0.001 |
| Glycosylated hemoglobin | ＜0.001 | ＜0.001 | ＜0.001 | 0.022 | 0.004 | 0.485 |
| eGFR | 0.019 | ＜0.001 | ＜0.001 | ＜0.001 | ＜0.001 | 0.713 |
| Chronic kidney disease | 0.241 | 0.003 | ＜0.001 | 0.064 | 0.001 | 0.168 |
| Hypertension | ＜0.001 | ＜0.001 | ＜0.001 | 0.024 | ＜0.001 | 0.009 |
| Family history of diabetes | 0.192 | 0.041 | 0.346 | 0.455 | 0.025 | 0.003 |
| Outcome diabetes^1^ | 0.459 | 0.056 | 0.005 | 0.009 | ＜0.001 | 0.376 |

^1^Outcome diabetes was defined as a subject who did not have diabetes at baseline but developed diabetes at follow-up.
